# The impact of adipose tissue distribution on endometrial cancer: a systematic review

**DOI:** 10.3389/fonc.2023.1182479

**Published:** 2023-05-29

**Authors:** Anouk A. S. van den Bosch, Johanna M. A. Pijnenborg, Andrea Romano, Bjorn Winkens, Louis J. M. van der Putten, Roy F. P. M. Kruitwagen, Henrica M. J. Werner

**Affiliations:** ^1^ Department of Obstetrics and Gynecology, GROW-School for Oncology and Reproduction, Maastricht University Medical Centre, Maastricht, Netherlands; ^2^ Department of Obstetrics and Gynaecology, Radboudumc, Nijmegen, Netherlands; ^3^ Department of Methodology and Statistics, Maastricht University, Maastricht, Netherlands

**Keywords:** endometrial cancer, adipose tissue distribution, prognosis, obesity, visceral adipose tissue, subcutaneous adipose tissue

## Abstract

**Introduction:**

Endometrial cancer (EC) is the most common gynecological cancer with a rising incidence, attributed to advanced life expectancy and obesity. Adipose tissue (AT) is an important endocrine organ, and its metabolic activity is affected by the different anatomical distribution or locations. AT distribution influences a number of diseases. In EC, it remains unclear whether the type of AT distribution affects development or prognosis. This systematic review aimed to determine whether AT distribution is associated with patient characteristics, disease characteristics, and patient prognosis in EC.

**Materials and methods:**

A search was conducted in Medline, MEDLINE EMBASE, and Cochrane Library. We included studies that enrolled patients with EC with any histological subtype and that distinguished between the visceral and subcutaneous AT compartment. In eligible studies, correlative analyses were performed for all outcome measures and AT distribution.

**Results:**

Eleven retrospective studies were included, with a wide range of measurements for the visceral and subcutaneous AT compartments. AT distribution was found to be significantly correlated to a number of relevant (disease) characteristics including obesity measures, histological subtype, lymph node metastasis, and sex steroid levels. Five studies reported on survival parameters including overall survival, progression-free survival and disease-specific survival, and they found that increased VAT volume was statistically significantly associated with a worse survival.

**Discussion/conclusion:**

This review demonstrates that there are significant correlations between AT distribution and prognosis, body mass index, sex steroid levels, and disease characteristics like histology. Well-designed, prospective, and larger-scale studies are needed to pinpoint these differences more specifically and understand how it can add in prediction and even therapy in EC.

## 1 Introduction

Endometrial cancer (EC) is the sixth most common cancer type in women worldwide with a rising incidence (1). Advanced life expectancy and obesity are the most important contributing factors for these increasing numbers (2). Obesity is defined as a body mass index (BMI) above 30 kg/m^2^ (3). Obesity is linked to a number of diseases like cardiovascular disease (CVD), diabetes, and hypertension (4, 5). It is also a risk factor for the development of multiple cancer types, with the strongest association for EC (6). Every five BMI units above the normal range (18–25 kg/m^2^) result in a 50% increase risk of developing EC (7). The association between obesity and EC is complex and only partially explained by the increased levels of circulating sex-steroid hormones in obese women. This may underlie that, despite this strong relationship of obesity with EC, the effects of obesity on EC characteristics and patient prognosis are still not fully understood. This includes the exact (molecular) mechanisms through which obesity facilitates EC development and understanding why (morbid) obesity does not cause EC in all women. In addition, it might clarify how obesity contributes to the rising incidence of non-endometrioid ECs, considered to be non-hormone sensitive (8). Furthermore, the impact of obesity on the prognosis of EC remains conflicting, as most patients with EC die because of CVD or other underlying comorbidities instead of EC (9). Three main hypotheses link obesity to cancer development: endogenous sex-steroid production, chronic hyperinsulinemia, and systemic inflammation (10, 11).

Adipose tissue (AT) is an endocrine organ that plays an important role in the production of a plethora of bioactive molecules with endocrine, paracrine, and autocrine functions (12). It has distinct metabolic activities depending on its anatomical locations. After menopause, circulating estrogens are produced predominantly in subcutaneous AT (SAT) through the conversion of androgens by aromatase (13). This mechanism of increased endogenous sex-steroid hormone production plays an important role in the development of EC, especially the endometrioid subtype. In contrast, visceral AT (VAT) plays a role in low-grade systemic inflammation and insulin resistance (14, 15), which have also been linked to cancer development.

Obesity is classified by the WHO as an abnormal or excess fat accumulation impairing health and includes any BMI ≥ 30 kg/m^2^ (16). BMI is a simple and clinically easily applicable indicator; however, it neither does discriminate muscle from AT nor does give insight in the AT distribution. Magnetic resonance imaging (MRI) and computed tomography (CT) perform equally well in visualizing and measuring AT distribution, including in subcutaneous, visceral, and intramuscular compartments (17).

The relationship between AT distribution and prognosis of CVD and, e.g., (colo)rectal cancer has been studied (18–22). However, the impact of AT distribution on EC characteristics, like FIGO stage, histology, and patient’ prognosis, is still unclear despite its tight relation with obesity. This systematic review aims to determine whether AT distribution is associated with patient characteristics (BMI and sex steroid levels), disease characteristics (FIGO, histopathology, and lymph node status), and patient prognosis.

## 2 Methods and materials

### 2.1 Study design and search strategy

We used the PRISMA 2020 checklist as a guideline to write this review (23). A search was conducted in Medline (1976 to May 2022), MEDLINE EMBASE (1951 to May 2022), and Cochrane Library, Database of Systematic Reviews for articles concerning this question (research question and search terms can be found in Supplementary File 1). The search strategy was constructed at the Maastricht University Medical Centre (MUMC+) by the primary researcher AvdB with support of a senior librarian of the Maastricht University.

**Figure 1:**
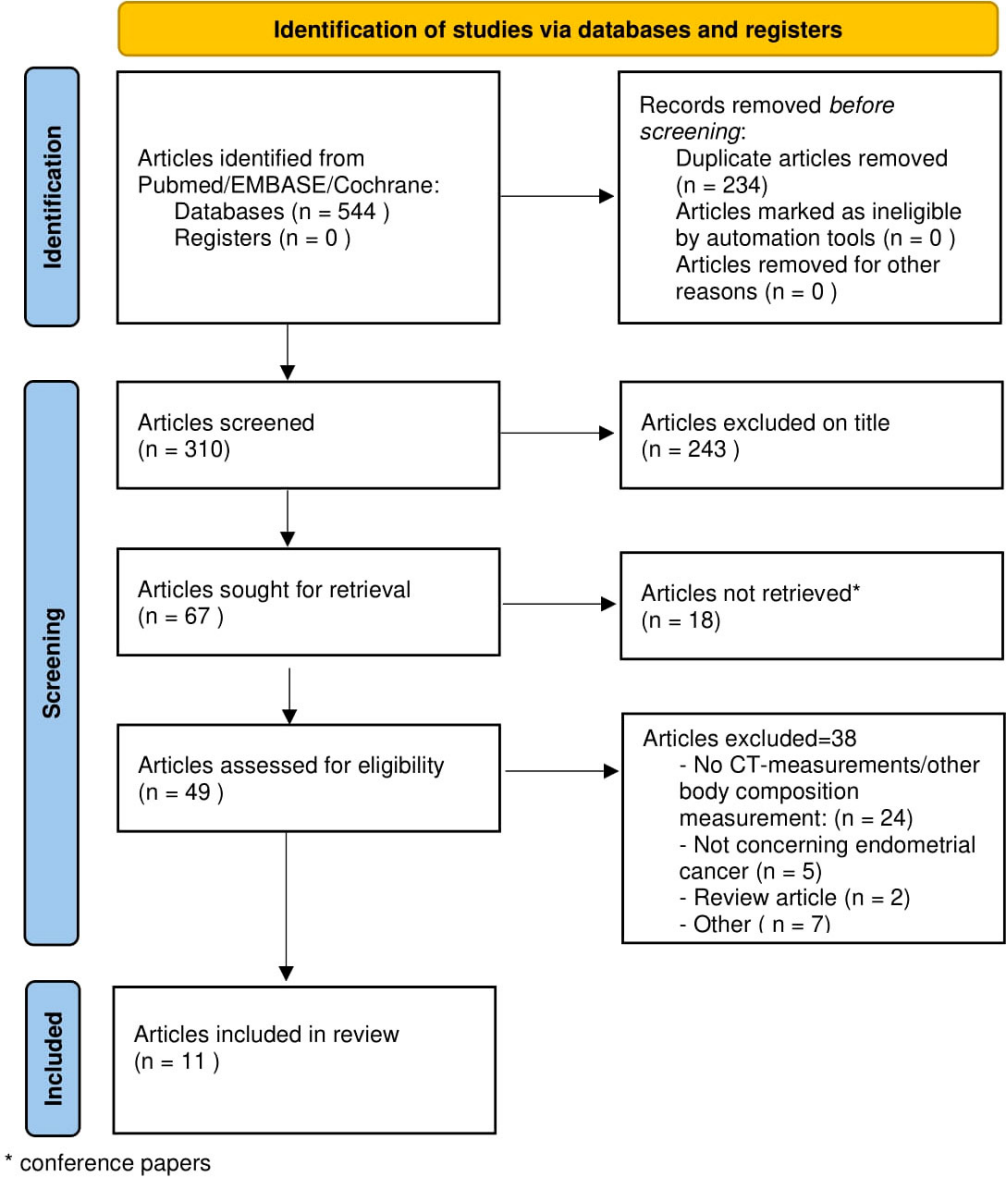
PRISMA flowchart of the selection of articles.

Our search was finalized May 2022. As far as possible, search terms were identical in the three databases to ensure comparable output. The search resulted in 310 hits (see **Figure 1**).

### 2.2 Selection of studies

Articles were included if they met the following criteria: articles should investigate the relationship between EC and visceral/subcutaneous (V/S) AT and meet the search criteria.

For this review, we included primary research papers, both of prospective and retrospective nature. We included studies that enrolled patients with EC with any histological subtype that distinguished between the visceral AT and SAT compartment, either through CT or MRI. Studies were excluded if the language was other than English, Dutch, or German. From all relevant articles, full text could be obtained. Because of a lack of a gold standard, all levels of measuring AT distribution (L3 through S1) were accepted. If studies did not report on all outcomes, they were included for the reported outcomes only.

Exclusion criteria: conference papers

### 2.3 Quality assessment

**Figure 2:**
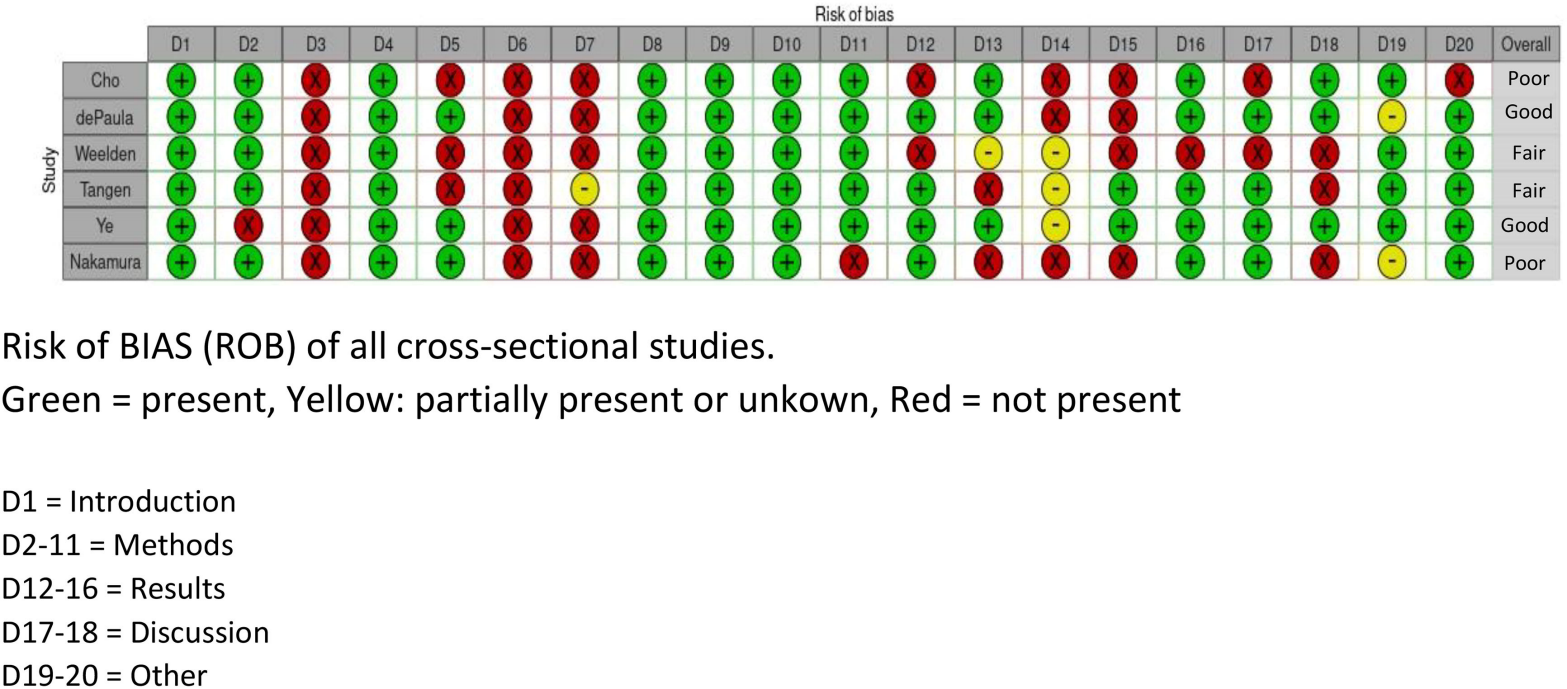
Risk of bias table, AXIS.

**Figure 3:**
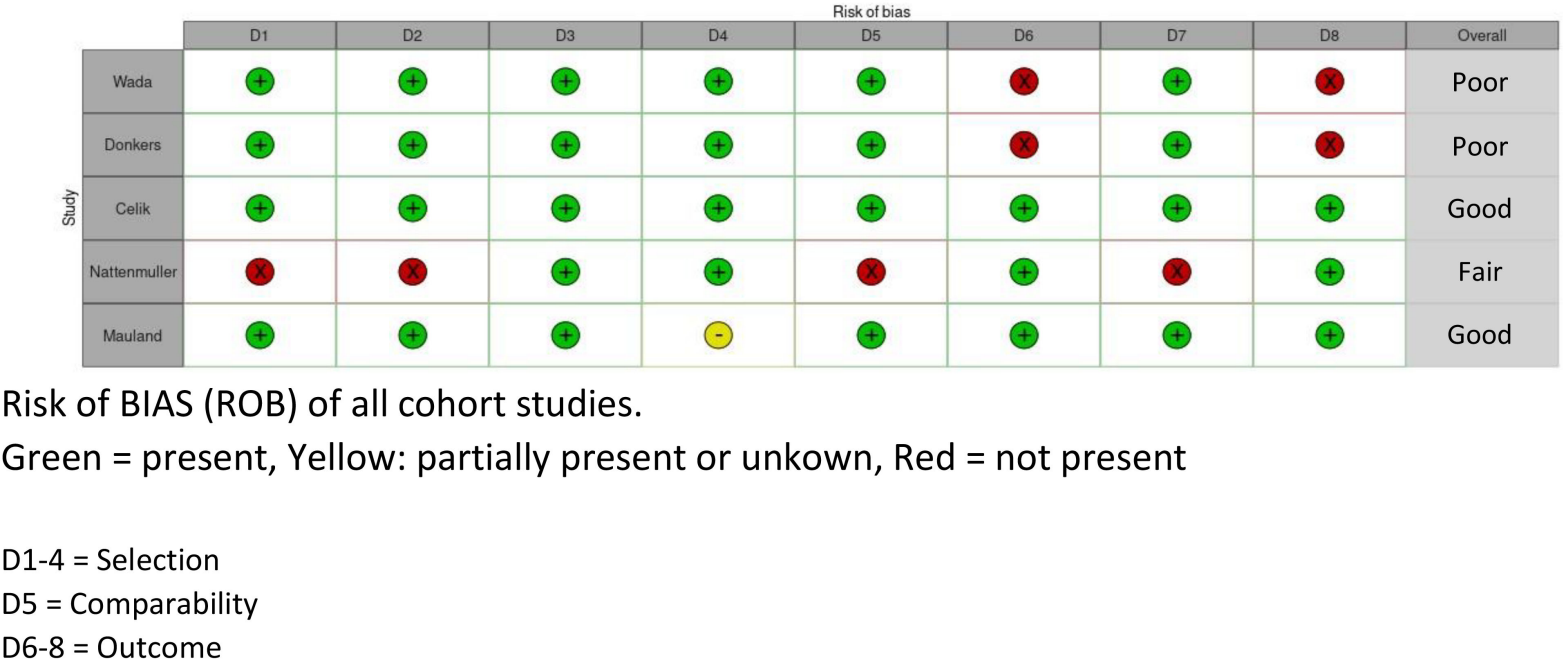
Risk of bias table, NOS.

To assess the risk of bias of the included studies, two different risks of bias tools were used to account for both cohort studies [Newcastle–Ottawa Scale (NOS)] and cross-sectional studies [appraisal tool for cross-sectional studies (AXIS)] (24, 25) (see **Figures 2**, **3**). The NOS has thresholds to convert the study assessment into a categorical scale of “good”, “fair”, or “poor”. The AXIS is more subjective in nature. To make the assessment more comparable, it was also converted to the previously mentioned scale. All scores were reviewed by two experts (AvdB and HW). Subdomains were scored separately and divided into three categories: good (if > 2/3 of the items were present and deemed acceptable), fair (if at least 1/2 of the items was present and deemed acceptable), or poor (if less than 1/2 of the items was present and deemed acceptable).

### 2.4 Outcome

We defined our primary outcome as the association of the type of AT distribution with patient characteristics and disease characteristics. The included patient characteristics consisted of BMI and sex steroid hormone levels; the disease characteristics were FIGO stage, histology, grade, myometrial invasion, tumor size, and lymph node status. As a secondary outcome, we aimed to determine the relationship between AT distribution and patient prognosis defined as (disease-specific/overall) survival. Meta-analysis was not possible after consulting a statistician (predominantly) due to heterogeneity in the quantification in AT compartments measurement in the included studies.

## 3 Results

### 3.1 Data extraction and characteristics of eligible studies

The PRISMA flow chart is shown in **Figure 1** and resulted in a total of 11 studies that fulfilled the inclusion criteria. Articles were published between 2011 and 2022. From these 11 articles, the following information was recorded: author, year of publication, journal, number of included patients, setting (university/teaching hospital/community hospital), EC subtype, FIGO stage, grade, mean age, mean BMI, AT measurements, level of imaging, and primary outcome and results (see **Table 1**). As shown in **Table 1**, the transverse CT plane of imaging that was used to measure the AT compartments was different between the studies that were included.

**Table 1:** Study characteristics of included studies.

Author	Year	Journal	Included patients	Hospital of inclusion	Type of endometrial cancer	FIGO	Grade	Mean Age	Mean BMI	Adipose tissue measurements	Unit	Imaging	Aim	Results
Nakamura	2011	Oncology reports	122	University Hospital, Okayama	All	All	I - 50% II - 20.5% III - 16.4%	56.98	X	VFA, SFA, TFA *	cm²	CT L4/L5	Determine fat accumulation in visceral and subcutaneous adipose tissue on CT. Study the relationship of these findings with clinical variables in the various histological types.	Patients with type I endometrial cancer have a statistically significant association with obesity-related biological parameters.
Donkers	2021	European journal of Obstetrics & Gynecology and Reproductive Biology	176	Royal Cornwell Hospital Trust, UK (academical hospital)	All	All	III - 100%	70.0	29.4	SAV, VAV, TAV *	cm³	CT L5/S1	Investigate the relationship between body fat distribution, assessed by CT-scan, in relation to overall and disease-specific survival in high-grade (grade 3) endometrial cancer patients.	In non endometrioid endometrial cancer, high visceral fat percentage was an independent predictor of poor survival. Hypertension and diabetes mellitus were significantly associated with high BMI and high visceral fat percentage.
dePaula	2020	Nutrition	545	Leading cancer institute, Brazil	All	All	I - 16.1% II - 25.1% III - 58.8%	64.5	29.8	SATI, VATI, SMI *	cm²/m²	CT L3	Provide the percentiles of distribution of body composition parameters according to cancer staging and body mass index (BMI). Identify the contribution of age, BMI, and cancer staging in the variation of the different parameters of body composition.	BMI was associated with body fat parameters and low-radiodensity SM index. Cancer stage was associated with SM index, mean SMD, and high-radiodensity SM index.
Ye	2016	BMC Cancer	200	Shanghai	All	I-III	I - 43.0% II - 42.5% III - 14.5%	54	24.7	VAT, SAT *	%	CT L4/L5	To assess the effect of visceral adiposity on clinical and pathological characteristics in patients with endometrial cancer.	Viscerally obese patients were more likely to be old and have positive lymph nodes as well as extrauterine disease.
Tangen	2019	Gynecologic Oncology	20	Haukeland University Hospital, Bergen	Endometrioid/ non-endometrioid	I/II	I/II - 50% III - 50%	X	25.2	VAV, SAV *	cm³	CT L5/S1	Investigate the relation between level of steroids in blood and prognosis for endometrial cancer patients.	DHEA, DHEAS, progesterone, 21 OH progesterone and E1S were significantly increased in patients with long survival compared to patients with short survival. Estradiol levels were significantly positively correlated with visceral fat percentage.
Nattenmüller	2018	Oncotarget	54	University Hospital Heidelberg	X	All	X	X	28.4	TAT, VAT, SAT *	cm²	CT L3/L4	Investigate the impact of body composition on overall survival (OS) in gynecological malignancies.	There was no statistically significant impact of any BC-parameters on OS.
Weelden	2019	BMC cancer	39	Radboudumc, Nijmegen (academical hostpital)	All	All	I - 10% II - 41% III - 48%	68.0	26.9	SAV, VAV, TAV *	cm³	CT L5/S1	Explore the relation between BMI, visceral and subcutaneous fat volumes and sex steroids and lipids levels in endometrial cancer patients.	Serum estradiol is moderately correlated with BMI and VAV and strongly correlated with SAV. Other sex steroids and lipids have weak and moderate correlations with VAV or SAV
Celik	2021	Obstetrics and Gynaecology Research	186	Istanbul University Institute of Oncology	Endometrioid/ non-endometrioid	All	I - 38.7% II/III - 61.3%	62.9	32.9	VAT, SAT *	cm²	MRI umbilical	Explore the relationship between VAT/SAT and survival in endometrial cancer patients.	Visceral adipose tissue is a significant and reliable prognostic indicator for endometrial cancer prognosis.
Mauland	2017	oncotarget	227	Haukeland University Hospital, Bergen	Endometrioid/ non-endometrioid	All	I/II - 68% III - 32%	66.9	27.9	SAV, VAV, TAV *	ml, %	CT L5/S1	Explore CT-quantified abdominal fat volumes and fat distribution in relation to BMI, clinicpathological features and survival in endometrial cancer patients.	High VAV% independently predicts reduced survival in EC patients.
Cho	2020	biomedical	52	Soonchunhyang University College of Medicine, Seoul	All	All	X	X	X	VFA, SFA, TFA *	cm²	CT L4/L5	Predict the effect of subcutaneous and visceral fat on endometrial cancer.	Unlike subcutaneous fat, visceral fat is more directly related to the development of endometrial cancer.
Wada	2022	International journal of clinical oncology	148	National Hospital Organization Kyoto Medical Center, Kyoto?	Endometrioid/ non-endometrioid	All	X	61.5	23.5	Visceral fat, Subcutaneous fat, V/S ratio	cm²	CT umbilical	Investigate the association between prognostic factors of type 1 and 2 endometrial cancer and obesity parameters.	A V/S ratio > 0.5 is a possible factor for poor prognosis in type 1 endometrial cancer.

Five cohort and six cross-sectional studies were included. Seven studies were retrospective and four prospective. The number of participants in these studies ranged from 20 to 545. Ten studies used CT imaging, and one MRI to quantify visceral AT and SAT. Four studies included women from Asian ethnicity, six studies included women from European populations, and one study included South American women. All studies but one focused solely on EC, whereas this latter focused on gynecological cancers and did perform subanalyses for patients with EC. Three studies included ≥ 50% women with high-grade (grade III) EC. Four studies included > 50% low-grade (grade I/II) tumors, and, in the remaining four studies, the subdivision was not clear. Furthermore, the BMI distribution was not equal in all studies, and mean BMI ranged from 23.5 to 32.9 kg/m^2^.

**Figure 4:**
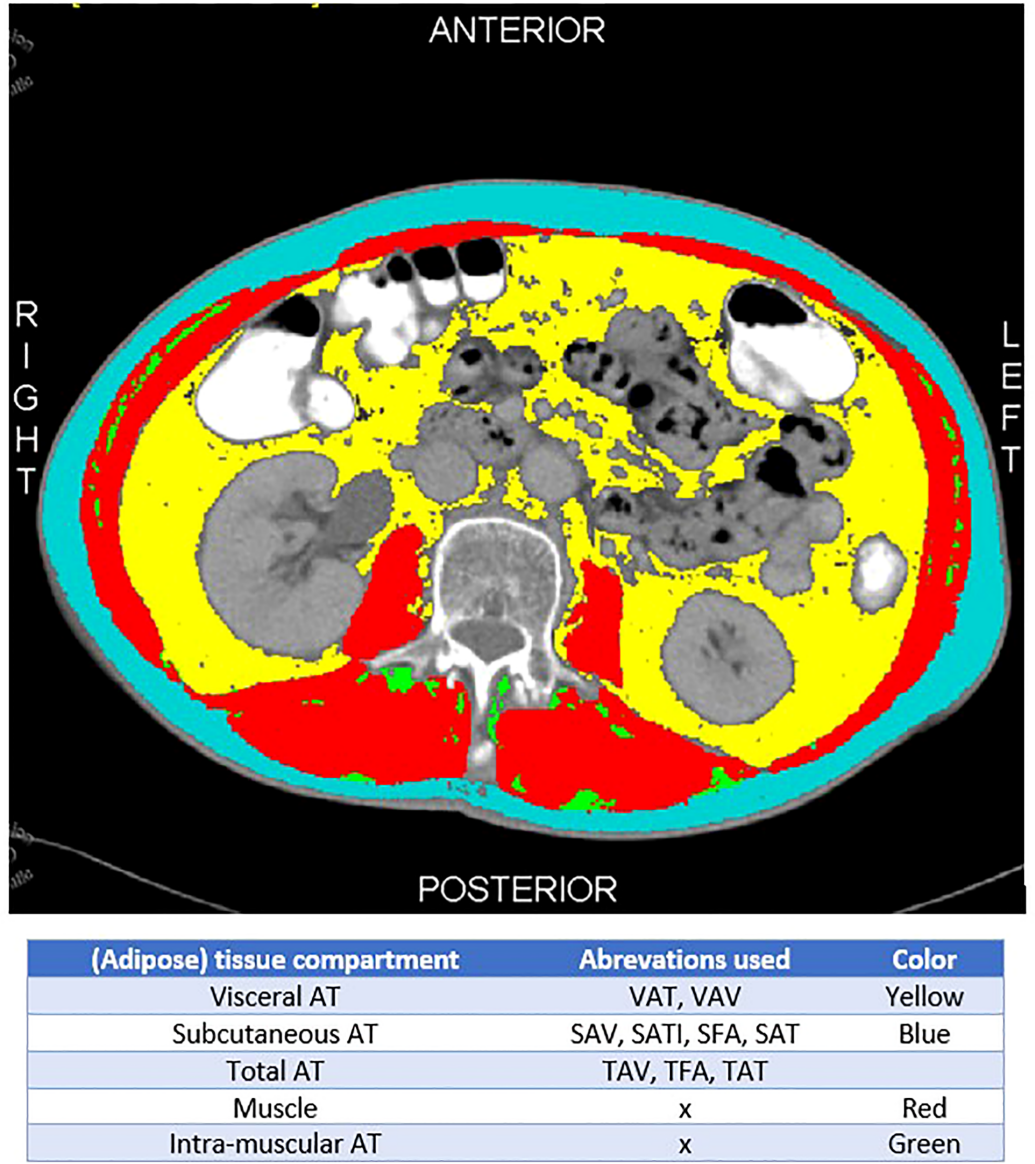
Explanation of AT distribution and different terminology.

All included studies investigated AT compartments on CT scan; however, different terminologies were used to describe the same AT compartments (see **Figure 4**). To facilitate legibility for the reader, we added **Figure 4**.

There was considerable variation in the quality of the included studies. Four studies were scored as “poor” quality (26–29), three studies were scored as “fair” (14, 30, 31), and four studies were scored as “good” quality (32–35). The reason for judging a study as “poor” was mostly due to lack of information in the methods and the results/outcome sections (see **Figures 2**, **3**).

#### 3.1.1 Relationship between AT distribution and patient characteristics

##### 3.1.1.1 BMI

Five studies (n = 746) explored the correlation between BMI and CT scan–based AT distribution (28, 29, 34–36). All five studies found a significant positive correlation between AT distribution and BMI (**Table 2**) (28, 29, 34–36), indicating that patients with a higher BMI also demonstrated higher quantities of AT on their CT scan. This relationship was significant for all measured AT distribution parameters as applied in different studies, including visceral, subcutaneous, and total AT (TAT). The two studies that investigated the relation between BMI and V/S ratio and VAT% did not find a significant relation between these parameters (29, 35).

**Table 2:** Relationship between adipose tissue (AT) distribution patient characteristics (BMI and sex steroid levels).

Relationship between AT distribution and BMI	Patients (n)	VFA/VAV	SFA/SAV	TFA/TAV	V/S ratio	VAT%
Cho	52	r^2 ^= 0.299 **p ≤ 0.0001**	r^2 ^= 0.528 **p ≤ 0.0001**	r^2 ^= 0.584 **p ≤ 0.0001**	x	x
Wada	145	R = 0.678 **p ≤ 0.01**	R = 0.872 **p ≤ 0.01**	R = 0.871 **p ≤ 0.01**	R = 0.05 p = 0.52	x
Ye	200	x	x	R = 0.667 **p ≤ 0.0001**	x	R = 0.743 p = 0.495
Nakamura	122	R = 0.743 **p ≤ 0.0001**	R = 0.895 **p ≤ 0.0001**	R = 0.907 **p ≤ 0.0001**	x	x
Mauland	227	r = 0.78 p ≤ 0.0001	r = 0.87 p ≤ 0.0001	r = 0.89 p ≤ 0.001	x	x
Relationship AT distribution and sex steroid levels	Patiënts (n)	VAV	SAV	TAV	BMI	VAV%
**Tangen**	20					
* E2		r = 0.42 p = 0.068	r = 0.005 p = 0.98	r = 0.24 p = 0.31	x	r = 0.47 p = 0.035
**Weelden**	39					
* E2		r = 0.58 **p ≤ 0.01**	r = 0.74 **p ≤ 0.01**	r = 0.74 **p ≤ 0.01**	r = 0.62 **p ≤ 0.01**	r = −0.06 NS
* A4		r = 0.29 NS	r = 0.43 **p ≤ 0.01**	r = 0.37 **p ≤ 0.05**	r = 0.26 NS	r = −0.17 NS
* DHEAS		r = 0.3 **p ≤ 0.05**	r = 0.3 **p ≤ 0.05**	r = 0.30 NS	r = 0.36 **p ≤ 0.05**	r = −0.10 NS

VFA, visceral fat area; VAV, visceral abdominal fat area; SFA, subcutaneous fat area; SAV, subcutaneous abdominal fat area; TFA, total fat area; TAV, total abdominal fat area; VAT%/VAV%, percentage of visceral adipose tissue; A4, androstenedione; DHEAS, dehydroepiandrosteronsulfate; x, outcome not reported; NS, not significant. Bold values are statistical significant values.

##### 3.1.1.2 Sex steroid hormone levels

Two smaller studies (n = 20 and n = 39) in postmenopausal women compared sex steroid hormone levels in relation to AT distribution (14, 31). Tangen et al., in a highly selective cohort of women with poor and good prognosis, reported a positive correlation between VAT percentage (VAV%) and estradiol (E2) levels (r = 0.47, p = 0.035; **Table 2**). Notably, neither BMI, TAT volume (TAV), SAT volume (SAV), nor VAT volume (VAV) were found to be significantly correlated with E2 levels (31). In contrast, Weelden et al., in a cohort selected on the basis of availability of a broad hormone analysis and preoperative CT scan, found a positive correlation between E2 and SAV (r = 0.74, p < 0.01), TAV (r = 0.74, p< 0.01), BMI (r = 0.62, p < 0.01), and VAV (r = 0.58, p < 0.01) (see **Table 3**). Androstenedione (A4) was positively correlated with SAV (r = 0.43, p < 0.01) and TAV (r = 0.37, p < 0.05). Dehydroepiandrosteronesulfate (DHEAS) was positively correlated with BMI, VAV, and SAV (r = 0.36, r = 0.35 and 0.34, all p < 0.05) (14).

**Table 3:** Relationship between adipose tissue (AT) distribution and disease characteristics (FIGO stage, histology, and other histopathological features).

Relationship between AT distribution and higher FIGO stage	Patiënts (n)	SATI/SAV		VATI/VAV	TAV	VAV%	HRSMI	BMI
de Paula	545	**p = 0.034**		p = 0.085	x	x	**p = 0.044**	x
Mauland	227	p = 0.66		p = 0.79	p = 0.90	p = 0.21	x	x
Donkers	176	p = 0.17		p = 0.45	p = 0.17	p = 0.88	x	p = 0.036
Relationship between AT distribution and histology (Type I and II endometrial cancer)	Patiënts (n)	VFA/VAV		SFA/SAV	TFA/TAV	VAV%	BMI	
Nakamura*	122	p = 0.309		**p = 0.005**	**p = 0.006**	x	**p = 0.006**	
Donkers	176	p = 0.64		p = 0.28	p = 0.88	p = 0.97	p = 0.66	
Relationship between AT distribution (VAT%) and histopathological features **	Patients (n)	Histology	Grade	Myometrial invasion depth	Tumor size	Positive lymph node status	LVSI	
Ye	122	p = 0.381	p = 0.069	p = 0.093	p = 0.791	**p = 0.042**	p = 0.582	

SATI, subcutaneous adipose tissue index; SAV, subcutaneous abdominal fat volume; SFA, subcutaneous fat area; VATI, visceral adipose tissue index; VAV, visceral abdominal fat volume; VFA, visceral fat area; TAV, total abdominal fat volume; TFA, total fat area; VAV%, percentage of visceral fat volume; HRSMI, high-radiodensity skeletal muscle index; BMI, body mass index; LVSI, lympho-vascular invasion; x, outcome not included in article. *, significant in type II EC; **, (VAT % < 31.89% and VAT% ≥ 31.89%). Bold values are statistical significant values.

#### 3.1.2 Relationship between AT distribution and disease characteristics

##### 3.1.2.1 FIGO stage

The relation of AT fat distribution and FIGO stage was reported in three studies including a total of 948 patients (27, 33, 34). The largest study (n = 545) observed a lower mean SAT index (SATI) in patients with a higher FIGO stage (FIGO stage III/IV) (p = 0.034) (33). Whereas, two other studies (n = 403 in total) did not find any significant association between AT distribution and FIGO stage [low (I/II) vs. high (III/IV)] (27, 34). These two studies included quite different patient populations, with 38% endometrioid EC and 100% grade III tumors in the study by Donkers et al. and 82% endometrioid EC with only 32% grade III tumors in the study by Mauland et al. However, a combination of these study characteristics was quite similar to that in the first study by de Paula et al.

##### 3.1.2.2 Histopathological characteristics

Two studies (n = 298) presented data on the relationship between AT distribution and histological subtype (27, 28). The first study, by Nakamura et al. (n = 122), that included predominantly grade I/II EC (>70%), observed that patients with endometrioid EC had a significant higher BMI (p = 0.006), increased subcutaneous fat area (SFA) (p = 0.005), and increased total fat area (TFA) (p = 0.006) when compared to patients with non-endometrioid subtypes (28). Donkers et al. (n = 176), who solely included grade III EC, however, did not find an association between any obesity parameters and endometrioid and non-endometrioid subtypes (27).

##### 3.1.2.3 Lymph node status

The study from Ye and colleagues was the only study reporting specifically on histopathological features in relation to VAT%. The study mostly included low-grade EC and only 14.5% high-grade EC. Higher VAT% in this study was significantly associated with the presence of lymph node metastases (p = 0.042), unrelated to subtype. They did not find any statistically significant association between VAT% and histological subtype, grade, myometrial invasion depth, tumor size, or lympho-vascular invasion (35).

#### 3.1.3 Relationship between AT distribution and patient prognosis

Five studies (n = 788), which were quite dissimilar in their patient cohorts, reported on survival parameters including overall survival (OS), progression-free survival (PFS), and disease-specific survival (DSS) (27, 29, 30, 32, 34). In two studies, the VAV% in relation to OS was evaluated (see **Table 4**). Mauland et al. (n = 227), with 82% endometrioid EC and 32% grade III tumors in their cohort, found that a VAV% ≥ 37% was independently associated with a reduced OS (p = 0.005) (34). Donkers et al. (n = 176), including 38% endometrioid EC and 100% grade III tumors, observed a similar relationship, but with a different cutoff value (VAV% > 34%) and only in univariable analysis. However, in subgroup analysis within non-endometrioid patients in the Donkers study, this association remained significant in the multivariable analysis for OS (p = 0.006) and DSS (p = 0.026) (27).

**Table 4:** Relationship between adipose tissue (AT) distribution and survival.

Relationship between AT distribution and Survival	Patients (n)	Patient group	Fat distribution parameter	Outcome	p-value
Mauland	227	All patients	VAV% ≥ 37%	Reduced OS (#)	**0.005**
Donkers	176	All patients	VAV% > 34%	Reduced OS ($)	**0.006**
		Non-endometrioid		Reduced OS & DSS (#)	**0.026**
Celik	186	All patients	VAT index > 0.265	Reduced DSS ($)	**0.029**
Wada	145	Endometrioid	V/S ratio (> 0.5)	Reduced OS ($)	**0.005**
				Reduced PFS ($)	**0.008**
Nattenmuller	54	All patients	Any	No effect on OS ($)	NS

#, multivariable analyses; $, univariable analyses; VAV%, visceral fat percentage; VAT index, visceral adipose tissue index; V/S ratio, visceral/subcutaneous index; OS, overall survival; DSS, disease-specific survival; PFS, progression-free survival; NS, not significant. Bold values are statistical significant values.

A third study, by Celik and colleagues (n = 186), classified patients into a VAT index ≤ 0.265 and a VAT index > 0.265. This index could not be translated to a clinical percentage based on the study information (32). This study, including a somewhat higher risk population with 61% grade III tumors despite 71% endometrioid EC, found no significant difference in PFS (p = 0.186); however, DSS was more favorable in the lower VAT index group (p = 0.029) (32). Wada et al. (n = 145), including a cohort with a relatively lower mean BMI of 23.5 kg/m^2^, explored the V/S ratio as a prognostic factor for PFS and OS in type I and II EC (29). The authors found that a V/S ratio > 0.5 was associated with a poor prognosis (OS and PFS) in univariable analyses including endometrioid (p = 0.0053 and p = 0.0080) but not in non-endometrioid EC (29). The remaining, smallest, study (n = 54) did not show a significant impact of AT distribution on OS (30). This study by Nattenmuller et al. also failed to provide any patients characteristics besides mean BMI.

## 4 Discussion

This review aimed to give an overview about the knowledge concerning AT distribution and EC. EC is considered to be affected by the obesity paradox, which presumes that, in contrast to an overall poorer prognosis due to obesity, obesity is associated with less aggressive biological subtypes of EC and, therefore, a better cancer specific prognosis may be found (9). However, this contrasts the observation that also the non-endometrioid or more aggressive subtypes show a rising incidence in obese women. As mentioned earlier, obesity is defined as a BMI above 30 kg/m^2^ (3). This definition, however, does not differentiate between the amount of AT or muscle or cover the complexity of AT distribution in visceral and subcutaneous compartments. Therefore one possible explanation for the obesity paradox is that it considers obesity as one entity and disregards these distinct localizations, subcutaneously or viscerally, with most likely different metabolic activity and distinct effects on cancer development. Low-grade inflammation is associated with VAT rather than with the SAT, where there is high aromatase activity. To our knowledge, this may distinctly affect EC development and fuel the attention for AT distribution and the way that we portray obesity (15).

Overall, this review had a number of notable findings that we will discuss in details. First, there is a strong correlation between BMI and imaging-based AT distribution measures. Second, studies indicate a significant association between AT distribution and sex-steroid hormone levels. Third, there are indications that a relation between AT distribution and histopathological findings exists. This relation is not consistent in the included studies, which may, in part, be explained by inclusion bias, as studies varied widely in subtypes and grades included. Last, and maybe most importantly, in all studies reporting about patient prognosis, increased VAV is associated with a worse survival (OS, DSS, and PFS) (27, 29, 32, 34).

All included studies found a significant positive correlation between BMI and the amount of SAT VAT and TAT (28, 29, 34, 36). BMI is the easiest way of classifying obese patients, and, currently, CT scans are not routinely performed for AT distribution (only). A study by Kammerlander et al. reported that simple anthropometric measures of obesity such as waist circumference and BMI were accurate for assessing cardiovascular risk in men but not in women. In women, VAT measurement through CT scan allowed a more precise assessment of obesity-associated cardiometabolic and cardiovascular risk (21). This underscores that there is an additional and clinical value in supplementing routine BMI measurement with more sophisticated measurements of other obesity-linked variables, including AT distribution above all in women. A similar study has not been yet carried out in patients with cancer.

Studying the relation between AT distribution and sex-steroid hormone level is challenging because of the uncertain contribution of pre- and postmenopausal ovaries to the systemic sex-steroid hormone levels. The retrospective nature of the included studies further complicates this. The two studies reporting on this outcome though included women with mean age of 66–68 and, therefore, presumably mostly postmenopausal women. Although sample size urgently needs to be enlarged, these studies demonstrate that AT distribution, specifically increased SAT and VAV%, is significantly associated with increased E2 levels. Future prospective larger studies are needed to confirm this relationship. We have recently set up the ENDOCRINE study, prospectively studying the effect of obesity, AT distribution, and oophorectomy on hormone levels in patients with EC and controls (37). This study may therefore be able to answer which AT compartment plays the most important role in E2 production and quantify how obesity and AT distribution contribute to differences in systemic sex-steroid hormone levels and resulting risk of EC.

The positive association between the higher amount of TAT and SAT and endometrioid type EC (28) fits with the classical etiological risk factors for endometrioid type EC (38). In the study by Nakamura, 70% of patients indeed suffered from low-grade endometrioid EC. This may therefore also support the lack of a similar association between AT distribution and subtype in the study by Donkers et al. (27), who only included high-grade EC, of which 60% of non-endometrioid subtype. The association between higher VAT% and a relative abundance of VAT with lymph node metastasis as reported by Ye et al. (35) may suggest a different and more aggressive tumor biology effect by VAT. Unfortunately, none of the other studies included lymph node metastasis as an outcome parameter. This more aggressive tumor biology might be in line with a study of Habanjar et al. They demonstrated that chronic low-grade inflammation resulted in a higher influx of macrophages in the tumor microenvironment, which stimulated angiogenesis, tumor cell motility, and infiltration. The macrophages also initiated the pre-metastatic site, promoting extravasation, survival, and sustained growth of tumor cells (39). Although speculative, as a higher amount of VAT results in a state of chronic low-grade inflammation, a higher incidence of lymph node metastasis may be expected (40).

Considering patient outcome, all studies reporting on this outcome demonstrated a worse prognosis, predominantly shown by a reduced OS and DSS, in patients with a higher VAV (27, 29, 32, 34). Relevant literature for comparison was mostly found in breast and colorectal cancer. A review in breast cancer by Picon-Ruiz et al. summarized that overall obesity was linked to both a shorter DSS and OS, both in pre- and postmenopausal women (41). Another breast cancer study focusing specifically on AT distribution found in their cohort a negative relation between the amount of SAT and OS but no relation between the amount of VAT and OS (42). This might be explained by the fact that patients with in the lowest VAT quartile were, on average, 12 years younger (48 years) compared with the patients in the highest quartile of VAT (60 years), affecting survival in itself. They also hypothesized that some parts of the abdominal SAT might have similar metabolic effects to VAT (41). However, this hypothesis has not been substantiated in other studies. A further study in (colo)rectal cancer in contrast showed a longer OS in patients with a higher SAT ratio but did not find VAT to be an independent prognostic factor (43). In a last study concerning colorectal patients, increased V/S ratio was significantly associated with a higher recurrence and shorter OS and DSS in patients with mid and low rectal cancer (22). These studies indicate that there is evidence on the role of AT distribution and survival in a number of cancer types. So far, there is evidence suggesting that AT distribution plays a role in the pathogenesis of several different cancer types. This evidence, however, is not conclusive yet and associations may be tumor specific.

There are a number of limitations that need to be addressed. First, studies used different measurements for displaying the AT distribution, like SAV, SAT, SATI, and SFA that are all used to display the amount of SAT. Using all these different terms makes the comparison and thus interpretation of these studies challenging (See **Figure 4**). Second, there is a plethora and heterogeneity in the quantification measures of the AT compartments in the included studies, precluding meta-analyses. For example, there is no agreement at what transverse CT-plane AT compartments are best measured. Because of the lack of a gold standard, all levels (L3 through S1) were accepted in this review but will need to be more standardized in future studies. In addition, this may have caused confounding in the results.

A broad search was performed to avoid missing any important studies in this research area. As a consequence, studies of moderate quality were also included, where varying degrees of selection bias were present, as documented in the risk of bias tables. This precluded strong conclusions.

To conclude, to our knowledge, this is the first review to summarize the evidence on the role of AT distribution on patient, disease characteristics, and prognosis in patients with EC. AT distribution may be the missing link between obesity and EC. There is strong evidence, already in these retrospective studies, that AT distribution affects patient prognosis in EC. Furthermore, correlations exist between AT distribution and patient and disease characteristics (including histology and lymph node status). Well-designed, prospective, and large-scale studies are essential to further understand and maybe find a way for more selective identification of women at risk of EC and even in therapeutic options for EC. Possible clinical applications might be improving the understanding of different drivers in the pathogenesis of EC and therefore develop a better tool in recognition of patients at risk and differentiate which patients would benefit from additional therapeutic options. Furthermore, specifying the role of obesity in the pathogenesis of EC supports educating the lay public in the importance of obesity prevention.

## Author contributions

AB - First authorship; HW: Equal contribution and last authorship; JP, AR, BW, LP, and RK: Equal contribution. All authors contributed to the article and approved the submitted version.
