# Supplementary material for: The impact of adipose tissue distribution on endometrial cancer: a systematic review
Source: Front Oncol. 2023 May 29;13:1182479. doi: 10.3389/fonc.2023.1182479 (PMC10258319; doi:10.3389/fonc.2023.1182479)
Supplement: Supplementary file 1 [file DataSheet_1.docx]

Supplementary file

**SEARCH**

Medline search: Intra-Abdominal Fat[MeSH] OR Subcutaneous Fat[MeSH] OR body fat*[tiab] OR ((intra-abdominal[tiab] OR intraabdominal[tiab] OR intra-peritoneal[tiab] OR intraperitoneal[tiab] OR subcutan*[tiab] OR visceral[tiab]) AND (adipos*[tiab] OR fat[tiab] OR obes*[tiab])) AND ("Endometrial Neoplasms"[Mesh] OR ((endometri*[tiab]) AND (neoplas*[tiab] OR tumor*[tiab] OR tumour*[tiab] OR cancer*[tiab] OR malign*[tiab] OR oncolog*[tiab] OR carcinom*[tiab])))

Embase search: exp intra-abdominal fat/ OR exp subcutaneous fat/ OR body fat*.ti,ab,kw. OR ((intra-abdominal.ti,ab,kw. OR intraabdominal.ti,ab,kw. OR intra-peritoneal.ti,ab,kw. OR intraperitoneal.ti,ab,kw. OR subcutan*.ti,ab,kw. OR visceral.ti,ab,kw.) AND (adipos*.ti,ab,kw. OR fat.ti,ab,kw.)) AND exp endometrium tumor/ OR ((endometri*.ti,ab,kw. AND (neoplas*.ti,ab,kw. OR tumor*.ti,ab,kw. OR tumour*.ti,ab,kw. OR cancer*.ti,ab,kw. OR malign*.ti,ab,kw. OR oncolog*.ti,ab,kw. OR carcinom*.ti,ab,kw.)))

Cochrane: MeSH descriptor: [Intra-Abdominal Fat] explode all trees OR MeSH descriptor: [Subcutaneous Fat] explode all trees OR MeSH descriptor: [Adipose Tissue] explode all trees OR ((intra-abdominal OR intraabdominal OR intra-peritoneal OR intraperitoneal OR subcutan* OR visceral) AND (adipos* OR fat)) AND MeSH descriptor: [Endometrial Neoplasms] explode all trees OR (endometri* AND (neoplas* OR tumor* OR tumour* OR cancer* OR malign* OR oncolog* OR carcinom*))
